# Topical Wound Healing Activity of Myricetin Isolated from *Tecomaria capensis* v. *aurea*

**DOI:** 10.3390/molecules25214870

**Published:** 2020-10-22

**Authors:** Abdelsamed I. Elshamy, Naglaa M. Ammar, Heba A. Hassan, Walaa A. El-Kashak, Salim S. Al-Rejaie, Ahmed M. Abd-ElGawad, Abdel-Razik H. Farrag

**Affiliations:** 1 Chemistry of Natural Compounds Department, National Research Centre, 33 El Bohouth St., Dokki, Cairo 12622, Egypt; wa.abdelaziz@nrc.sci.eg; 2 Therapeutic Chemistry Department, National Research Centre, 33 El Bohouth St., Dokki, Cairo 12622, Egypt; nm.ammar@nrc.sci.eg (N.M.A.); ha.el-saud@nrc.sci.eg (H.A.H.); 3 Department of Pharmacology & Toxicology, College of Pharmacy, King Saud University, Riyadh 11451, Saudi Arabia; rejaie@ksu.edu.sa; 4 Plant Production Department, College of Food & Agriculture Sciences, King SaudUniversity, P.O. Box 2460, Riyadh 11451, Saudi Arabia; 5 Department of Botany, Faculty of Science, Mansoura University, Mansoura 35516, Egypt; 6 Departments of Pathology, National Research Centre, 33 El Bohouth St. Dokki, Cairo 12622, Egypt; ar.hussein@nrc.sci.eg

**Keywords:** myricetin, flavonoids, wound healing, *Tecomaria capensis* v. *aurea*, inflammatory cytokines

## Abstract

Wounds and burn injury are major causes of death and disability worldwide. Myricetin is a common bioactive flavonoid isolated naturally from the plant kingdom. Herein, a topical application of naturally isolated myricetin from the shoots of *Tecomaria capensis* v. *aurea* on excisional wound healing that was performed in albino rats. The wounded rats were treated every day with 10 and 20% myricetin for 14 days. During the experiment, the wound closure percentage was estimated at days 0, 7, and 14. Effects of myricetin on the inflammatory cytokines (tumor necrosis factor-*α* (TNF-*α*), interleukin-1*β* (IL-1*β*), and cluster of differentiation 68 (CD68) in the serum were evaluated using immunosorbent assay kits. The percentage of wound closure and contraction was delayed in wounded rats (67.35%) and was remarkably increased after treatment of wounded rats with myricetin; the treatment with 20% myricetin was the most potent (98.76%). Histological findings exhibited that 10% myricetin caused the formation of a large area of scarring at the wound enclosure and stratified squamous epithelium without the formation of papillae as in the control group. Treatment with 20% myricetin exhibited less area of scarring at the wound enclosure as well as re-epithelialization with a high density of fibroblasts and blood capillaries in the wound. Level elevations of serum pro-inflammatory cytokines, IL-1*β*, and TNF-*α* and macrophage CD68 were decreased in wounded rats treated with myricetin. Thus, it can be suggested that the enhancements in inflammatory cytokines as well as systemic reorganization after myricetin treatment may be recommended to play a crucial part in the promotion of wound healing. The findings suggest that treatment with a higher dose of myricetin was better in improving wound curing in rats. It could serve as a potent anti-inflammatory agent and can be used as an adjunctive or alternative agent in the future.

## 1. Introduction

Skin is the biggest organ in human beings and animal bodies, acting as the physical shield against harmful pathogens and radiation, and maintain life on a continuous basis. Trauma such as battle, biting, thermal burning, a car accident, and others may cause a break of the skin continuity and wounding [1,2]. Skin injury healing is a complicated and dynamic process that remains a major clinical issue. Various techniques have been proposed, including ointments, wound dressings, and grafts transplantation to guarantee tissue repair [3,4]. As synthetic drugs represent a high risk of side effects, including allergic reaction and drug resistance, natural compounds are becoming powerfully implied in alternative wound healing medicines [5]. Alternative herbal medicine involves moist wound healing, which does not require long-term dressing [6]. Some of the herbs have antimicrobial properties that can kill bacteria and avoid infection with wounds [7].

The ornamental plant, *Tecomaria capensis* v. *aurea* (family: Bignoniaceae), has been reported to have various traditional uses such as diarrhea treatment, enteritis, pneumonia, fragrance treatment, and tonic uses [8]. The different extracts of this plant have also been reported to exhibit antioxidant [9], antipyretic [10], antifungal, antimicrobial [8], and cytotoxic activities [11]. Chemically, the previous study showed that this plant is extremely rich in phenolic compounds, especially flavonoids [11].

**Figure 1:**
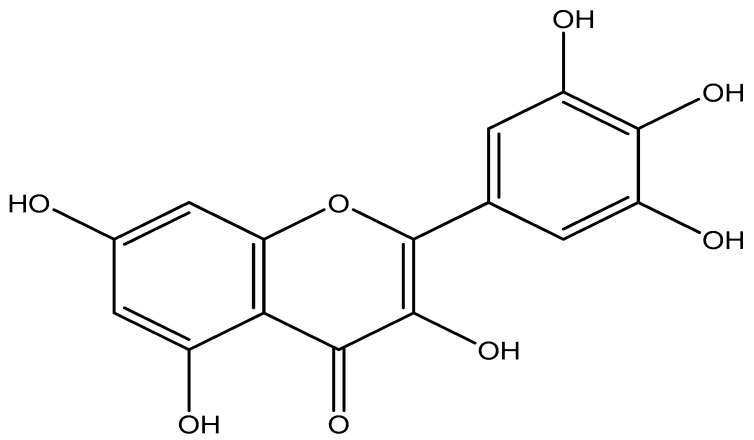
Chemical structure of myricetin isolated from *T. capensis.*

Flavonoids were the principal polyphenolic components of practically all medicinal plants [12,13]. It has been documented that flavonoids have antioxidant, anti-inflammatory, anticancer, cardioprotective, hepatoprotective, anti-diabetic, and antimicrobial effects [13]. Myricetin, a flavonoid compound generally found in medicinal plants (Figure 1), has been reported to have several potent biological actions, as anti-inflammatory, antidiabetic, antioxidant, anti-hypertensive, anti-allergic, analgesic, and immunomodulatory function [13,14,15,16]. In addition, myricetin’s anticancer effects (including antiproliferative, anti-invasive, and antiangiogenic) have been deduced from many forms of cancer such as colon, stomach, and ovarian cancer [17,18]. However, this compound’s function as a wound-healing agent has not been studied to date.

From this point of view and continuing the objectives of our team to identify effective biological agents from naturally isolated metabolites [19], the goals of this study are (i) the isolation and identification of the major flavonoid of isolated from *T. capensis*, myricetin, (ii) the in vivo study of wound healing activity of myricetin in a rat model as revealed via biochemical, histological, and histochemical examinations, and (ii) the establishment of the associated changes in inflammatory cytokines TNF-*α*, IL-1*β*, and the immunoglobulin CD68 levels (macrophage) during the wound healing process.

## 2. Results and Discussion

### 2.1. Wound Closure Estimation

The injured areas of the treated rats are shown in Table 1. After wounding, the repair process was tested in each group by comparing the wound area or wound closing on day 0 and day 14. There was no apparent difference between the groups on the first day. While on the 14th day, the wounds treated with different treatments reported a decline in wounds. The wound area assessment (Figure S1) showed that the topical use of myricetin-based cream substantially decreased wound area (Table 1), with a marked improvement in the lesion retraction rate compared to the positive control.

The wounded group showed a reduction in the percentage of wound contraction compared to the treated groups. The highest rate of wound contraction was observed in animals treated with 20% myricetin as compared with other groups at day 14 (Table 1). Therefore, a better evident effect was seen by the 20% myricetin treated animal.

### 2.2. Quantitative Analysis of TNF-α and IL1-β

The results, presented in Table 2, showed that the activity of the inflammatory markers TNF-*α* and IL1 *β*, respectively, was markedly upregulated in incisional wounded rats compared to the normal control rats. While wounded rats treated with dermazin, paraffin, and 10% and 20% myricetin displayed a high reduction in the inflammatory markers (TNF-*α* and IL-1*β*). Interestingly, 20% myricetin showed significantly decreased in TNF-*α* and IL-1*β* production at (*p* < 0.05), and was the most close to the normal control group.

### 2.3. Effect of Myricetin on CD68 Level in Experimental Rats

**Figure 2:**
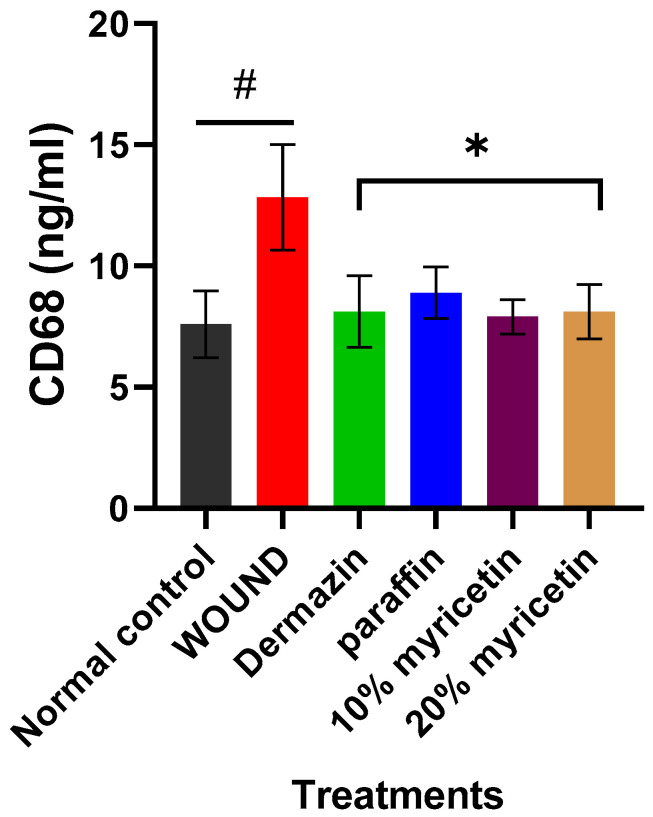
Effect of myricetin treatment on serum levels of CD68 in rats. * *p* < 0.05 versus wounded rats, **^#^***p* < 0.05 versus normal control.

Results of CD68 level in the wound skin of all experimental groups are presented in Figure 2. Wounded control rats showed an increase in CD68 level (*p* < 0.05) when compared to normal control rats. Conversely, in the wounded rats treated with dermazin, paraffin, and 10% and 20% myricetin, a significant reduction in CD68 level (*p* < 0.05) was noticed in respect to the wounded control rats. The results indicated that treatment with 10% and 20% myricetin was the most effective in the reduction of CD68 level compared to the positive group (Figure 2).

### 2.4. Histological Results

**Figure 3:**
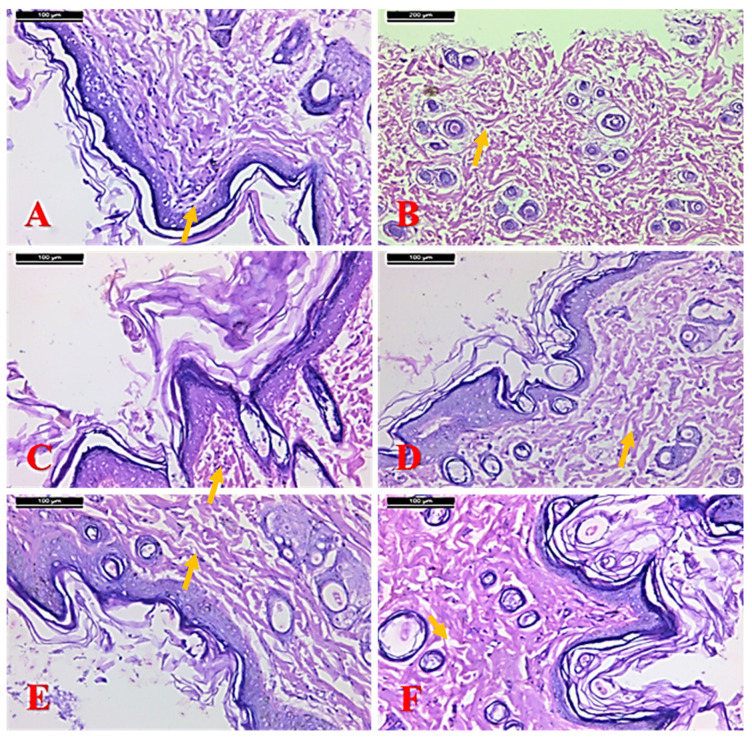
Photomicrographs of a skin section from (**A**) a control rat showing a well-formed thick granular cell layer, the dermis contains a deposit of collagen fibers and minimal inflammation, (**B**) control positive group, (**C**) 10% myricetin treated group, (**D**) 20% myricetin treated group, (**E**) drug-treated group, and (**F**) paraffin treated group (yellow arrows: inflammations) (H&E stain, scale bar 100 µm).

The histological examination of dorsal skin samples from rats of the control group revealed a normal structure of epidermis and dermis layers of the skin. The epidermis was composed of a thick layer of normal stratified squamous epithelial cells. The papillary layer of the dermis exhibited abundant capillaries and connective tissue cells, where the reticular layer of the dermis was composed (Figure 3). The histological evaluation of skin from the positive control rats illustrated degeneration and necrosis in the epidermis and degenerative changes in hair follicles (Figure 3B). In a microscopic examination of the skin samples from the 10% myricetin treated group, a large area of scarring at the wound enclosure was seen; as well as wounds were covered by stratified squamous epithelium without a formation of papillae as in the control group (Figure 3C). In the case of the 20% myricetin treated group, a reduced area of scarring at the wound enclosure was seen and re-epithelialization and a high density of fibroblasts and blood capillaries were observed in the wound (Figure 3D).

The findings of the drug-treated group showed a number of fibroblasts, macrophages, neutrophils, and collagen fibers (Figure 3E). In the paraffin treated group, stratified squamous epithelium without a formation of papillae, more scarring at the wound enclosure, and more collagen fibers were found (Table 3). Note the flattened basal layer covering the wounded area (Figure 3F).

**Figure 4:**
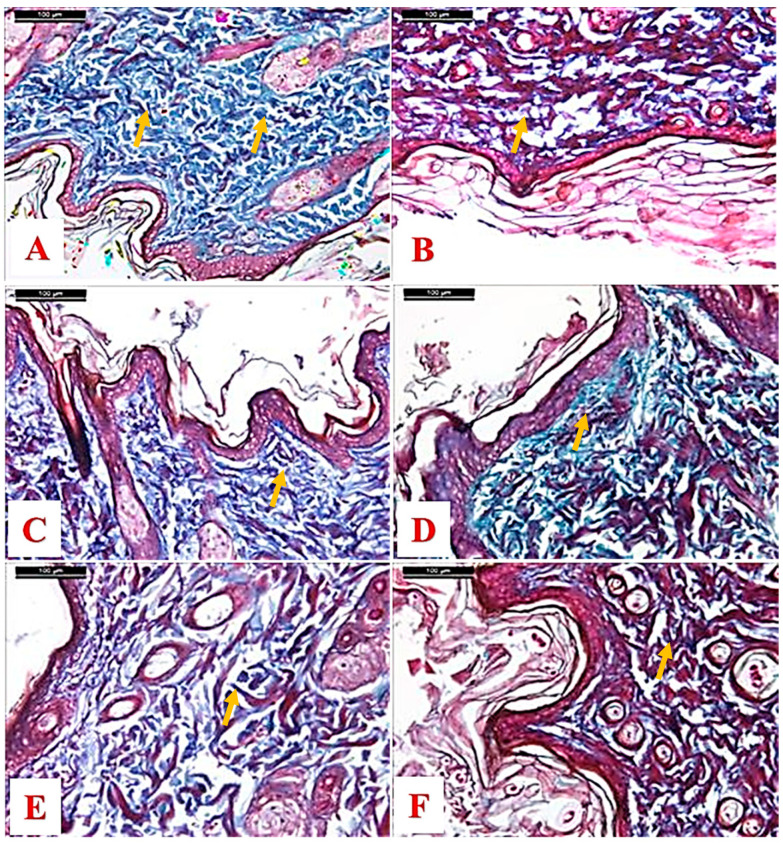
Photomicrographs of a skin section from (**A**) a control rat showing well-formed but thick granular cell layer, the underlying dermis contains deposited collagen fibers with minimal inflammation, (**B**) positive control group, (**C**) 10% myricetin treated group, (**D**) 20% myricetin treated group, (**E**) drug-treated group, and (**F**) paraffin treated group (yellow arrows: collagen deposit) (Masson’s trichrome stain, scale bar 100 µm).

In Masson’s trichrome, stain the density of collagen fibers appeared in the normal form and distribution in the dermis of control (Figure 4A, Table 2) but in the positive control a clear reduction was noticed compared with the control (Figure 4B). In the case of myricetin treated groups, 10% had a lesser increase than the 20% when compared with the positive control (Figure 4C,D). The drug revealed a more or less normal form and distribution (Figure 4E), while in the paraffin treated group the distribution is reduced when compared to the drug group (Figure 4F).

Wound healing typically starts following an injury and continues through the well-organized incorporation and relations of different pathways, growth factors, and various cell and tissue types. The wound healing pattern is defined by constricting the wound area via the central movement of the wound border towards the wound core, inducing wound closing. In the current study, wound healing was detected by morphological evaluation, and the measured percentage of wound healing was delayed in wounded rats compared to myricetin treatment. These results are in concordance with Gawish et al. [20] who reported that the wound area was elevated in the wounded group revealing delayed healing. The findings of the present study showed a marked elevation in serum levels of pro-inflammatory cytokines IL-1*β* and TNF-*α* levels in wounded rats, indicating an increase in inflammation. Studies found that dermal wound healing involves three phases: an inflammatory process due to pro-inflammatory mediators secretion and immune system suppression, proliferative phase via the proliferation of fibroblasts, collagen accumulation, and new blood vessels development, and a remodeling phase that includes regeneration and wounded tissue reconstruction [21,22,23]. The medications that accelerate wound healing with a possible contribution in all phases of the process will be needed for effective treatment, with low cost and fewer side effects.

The results of this study showed that treatment with myricetin for wounded rats significantly decreased the elevated levels of IL-1*β* and TNF-*α*. These results have led us to suggest that myricetin accelerates the switch from inflammatory to anti-inflammatory responses, which promotes curing. These findings are consistent with other studies showing increased levels of TNF-*α* and IL-1*β* in serum rats after a burn-injury [24,25].

In addition, TNF-*α* and IL-1*β* are inflammatory cytokines that show an important role in the early phase of the wound healing process [26,27]. While being included in the inflammatory process, proinflammatory cytokines are also used as mediators of cell proliferation and differentiation throughout the wound-healing phase. [28]. TNF-*α* has a dual role in decreasing the development of granulation tissue and the organization of collagen fibers [29]. In the present study, myricetin treatment positively normalized these mediators by decreasing pro-inflammatory cytokines such as TNF-*α* and IL-1*β*. 

Classically, (M1) and alternatively (M2) activated macrophages are important in regulating wound healing and tissue regeneration. These cells produce pro-inflammatory cytokines that kill pathogens and other foreign materials. These macrophages also produce diverse growth factors involved in controlling inflammation, restoring wound tissue, and healing [30]. CD68 is a glycoprotein and an indicator of wound healing macrophages. In our study, we observed a rise in CD68 levels in wounded rats (Group 2), which supported the concept of inflammation and, subsequently, phagocytic activity. The elevation of 10% and 20% of myricetin treated groups is reduced to a minimal level. The findings of our study are similar to those described by Li et al. [31] and Salim et al. [32] that showed an elevation in the expression of CD68 involved in wound healing; it appears that macrophages are widely distributed throughout the body and play a role in the inflammatory phase as the body’s response to foreign particles and bacteria. Wound healing is a very complicated process including multifactor events sequences with numerous biochemical and cellular procedures. The main targets of these procedures are to rejuvenate and rebuild the skin ruptured anatomical continuity and functional roles [33]. In this study, the results exhibited that myricetin caused the acceleration of healing and mending of the wound by covering the overall wound thickness area with an organized epidermis that supported by the existence of mature scar tissue. The documented potent anti-inflammatory activities of flavonoids, especially myricetin, support the capability of this compound to enhance the healing of wounds throughout the two macrophages M1 and M2 [34].

Polyphenolic compounds, especially flavonoids, are widely distributed constituents in the plant kingdom and described as potent anti-inflammatory and wound healing agents [35]. Due to their potent anti-phlogistic role, some flavonoids were found to have a significant role as wound healing agents in vitro and in vivo models such as apigenin, rutin, catechin [35,36], sativanone-7-O-glucoside, ononin [37], prunetin, genistein [38], luteolin, linarin, 6-hydroxyluteolin [39], kaempferol, quercetin, and vicenin-2 [40].

Wang et al. [14] reported that myricetin has a potent anti-inflammatory effect in xylene and carrageenan-induced rats via the (i) inhibition of paw edema, (ii) significant reduction of the levels of oxidative stress markers such as malondialdehyde (MDA) and superoxide dismutase (SOD), (iii) significant reduction of the leukocyte count, and (iv) inhibition of granuloma tissue formation through the inflammation. Concurrently, Lee and Choi [41] described the potent anti-inflammatory activity of myricetin via its significant role in the decreasing of the production of IL-1*β* and IL-6. Herein, our data were found in complete agreement with all these previous documented data that strongly confirmed the potent inhibitory role of myricetin in the production of inflammation markers. The glycoside derivatives of myricetin and myricetin-3-*O*-*β*-rhamnoside were described as having in vitro healing activity via promoting the migration of fibroblasts [42]. Additionally, myricetin has been reported to act in the suppression of the inflammation responsible factors and enzymes by inhibition of production of COX-1, COX-2, and TNF-*α* [34]. The two isolated compounds from *Ipomoea carnea*, kaempferol, and its 3-O-*β*-D-glucoside, were reported to have significant wound healing activities in Wistar rats at 200 mg/kg [43]. In another study, kaempferol was documented to have potent activity as a topical wound healing agent in diabetic and nondiabetic rats at different concentrations, especially at a concentration of 0.5% (*w*/*w*) [44]. Also, the wound closure was observed by Gopalakrishnan et al. [45] in wounded rats time-dependently using 1% ointment and 5% gel of quercetin [46].

Several theories were established that described the relationship between the anti-inflammatory activities of flavonoids and their structures [47]. All these theories were constructed basically upon the hydroxyl groups, especially in rings A and B. The presences of hydroxyl groups in C-5, C-7 in ring-A, and C-4′ in ring-B in flavonoid structures were found to have a main role in the significant inhibition of production of TNF-*α* [47], while the absence of the hydroxyl group at C-4′ was deduced to not reduce TNF-*α* production and may augment its production [47]. Previous evaluations suggested that the oral administration of flavonoids including the hydroxyl group in C-3′ in ring-B may have a minimal effect on TNF-*α* levels due to affecting the absorption or metabolism of the compound [48,49,50]. Therefore, the topical administration of myricetin was favorable, as described in the present work. Also, the published data stated that the topical administration of 5, 7, 3, 3′, 4′-pentahydroxy flavonoids encourage wound healing via enhancing collagen matrix formation and modulating of the cytokines levels [51]. Here, and in agreement with the previous studies, myricetin caused a time-dependent closure of the wound alongside a remarkable decrease of TNF-*α* production.

## 3. Materials and Methods

### 3.1. General Experimental Procedures 

NMR and LREI-MS experiments were performed using the JEOL JNM-ECA 600 spectrometer (JNM-ECA; Tokyo, Japan) and JEOL JMSGCMATE mass spectrometer (JNM-ECA; Tokyo; Japan), respectively. Chromatography and separation were performed using normal-phase silica-gel BW-200 (Fuji Silysia Chemical, Ltd., 150–350 mesh). The silica-gel thin layer chromatography (TLC) plates with spots were developed and heated with vanillin-H_2_SO_4_ spraying reagent.

### 3.2. Plant Materials Collection and Preparation

The shoots of *T. capensis* v. *aurea* were collected in March from the garden of Mansoura University, Mansoura, Egypt (31°02′29.1″ N 31°21′21.4″ E). The collection and identification of the plant were performed by Dr. Ahmed Abd-ElGawad, Associate Professor of Plant Ecology, Botany Department, Faculty of Science, Mansoura University. A voucher specimen (NRC-IX018-TC-1185) was deposited in the herbarium of the National Research Center, Egypt. The shoots were dried in air in a shaded place at room temperature (25 ± 3 °C) for ten days.

### 3.3. Extraction, Isolation, and Identification of Myricetin

The air-dried shoots of *T. capensis* (2250 gm) were extracted by 70% hydroalcoholic methanol for three days at room temperature. The extract was concentrated in vacuo to obtain a gummy residue (135 g). The concentrated crude extract was fractionated on silica gel flash CC (5 × 60 cm) and eluted with gradient solvents of increasing polarity starting with CHCl_3_-MeOH step-gradient afforded six main fractions (TC-1: TC-6). The fraction TC-3 was further chromatographed over Sephadex LH-20 using CHCl_3_-MeOH (1:2) afforded compound **1** (123.4 mg). Compound **1** was established as myricetin using a modern technique such as ^1^H NMR (Bruker, 500 MHz) and ^13^C NMR (125 MHz) as well as mass spectroscopy.

#### Physical Properties and Spectroscopic Data of Myricetin

Yellow powder, m.p. 356–358 °C (standard: 357 °C), *R_f_*: 0.57 (CHCl_3_-MeOH; 1:1), EI-MS, *m/z*: 319 [M + H]^+^, ^1^H NMR (600 MHz, CD_3_OD): *δ* 7.31 brs (H-2′, 6′), 6.47 d (*J* = 1.7 Hz, H-8), 6.18 d (*J* = 2.5 Hz, H-6). ^13^C NMR (125MHz, CD_3_OD): *δ* 176.0 (C-4), 164.3 (C-7), 161.0 (C-5), 156.2 (C-9), 146.9 (C-2), 145.7 (C-3′, 5′), 136.0 (C-3), 135.8 (C-4′), 120.6 (C-1′), 107.5 (C-2′, 6′), 103.1 (C-10), 98.3 (C-6), 93.4 (C-8). 

### 3.4. Experimental Animals

Adult female albino rats weighing 150 to 170 g were collected from the National Research Center’s Animal House Colony in Cairo, Egypt, and were acclimatized in a particular area of 25 ± 2 °C temperature and humidity 55%. Rats were constantly evaluated at the National Research Center Animal Facility Breeding Colony with 12 h of light/dark cycles. Rats were housed individually with ad libitum access to a normal laboratory diet and tap water. All animal procedures comply with the recommendations of the Experimental Animal Ethics Committee, National Research Centre, Cairo (Ethical Approval Number: 20-045), Egypt, as illustrated in the Guide to Care and Use of Laboratory published by the US National Institute of Health Policy. 

### 3.5. Ointment Preparation

The preparation of the used ointments occurred via the method of fusion according to the British Pharmacopoeia [18]. The ointments were prepared by mixing 10% and 20% (*w*/*w*) of myricetin and soft paraffin with good stirring. The two concentrations of the myricetin (10% and 20%) were selected based upon the previous studies of the similar compounds [52] as well as the previous biological activities of this compound itself [13,14,15,16]. Dermazin cream (silver sulfadiazine 1%) was used as a reference drug [53].

### 3.6. Methods Incisional Open Wound Study 

The current study included 36 albino female rats (150 to 170 g, six rats/group). Wound incision conception: under light ether anesthesia, shearing of the hair of the dorsal midline of rats was conducted. A longitudinal full-thickness wound incision 2 cm long was made in the dorsal midline through the skin and subcutaneous tissue down to the fascia of the subcutaneous muscle layer. The present study was organized on six groups of rats which were assigned as follows: group 1 (**G1**): normal control group, rats were kept without any incision wounds, group 2 (**G2**) positive control: rats with an induced surgical incision wound 2 cm long in the skin of the back of the animal left undressed. Group (G3, G4, and G5): rats with a surgical incision wound were treated topically with myricetin 10 and 20% (*w*/*w*), paraffin, and a standard drug (Dermazin cream (silver sulfadiazine 1%)), respectively, immediately after incision-wound creation. The myricetin (10 and 20%), dermazin, or paraffin were topically applied once a day in wounded rats for 14 days. Each group was sacrificed after 15 days from the start of the experiment [47].

### 3.7. Samples Collection

Macroscopic wound incision measurements, photographs, and the length of the wound incision were taken from rats in all classes. Blood samples from the rats in each group were then collected using the EDTA free tube orbital sinus technique to isolate sera for tumor necrosis factor-alpha (TNF-*α*), IL1*β*, and CD68 measurements. 

### 3.8. Measurement of the TNF-α and IL-1β Level in the Rat Serum

TNF-*α* and IL-1*β* levels in serum rats were measured using a commercial kit for an enzyme-linked immune sorbent assay (ELISA), as directed by the manufacturer. The cytokine concentrations were measured using a standard curve and expressed as Pg/mL [54].

### 3.9. Estimation of CD68 in the Rat Serum

CD68 level in serum rats was tested using a commercial kit for an enzyme-linked immune sorbent assay (ELISA) according to the manufacturer’s instructions. The level of the macrophage CD68 was measured using a standard curve and expressed as ng/mL.

### 3.10. Measurement of Wound Diameter and Closure

The wound diameter was measured after the incision on days 0, 7, and 14. The percentage of wound closure was estimated using the following: 

wound closure rate on the day X (percent) = ((wound diameter on the day 0 − wound diameter on the day X)/(wound diameter on the day 0)) × 100 [55].

### 3.11. Histopathological Examination 

Samples of dorsal skin were taken from all animals, fixed in buffered formalin, processed through a graded series of alcohol and xylene, and embedded in blocks of paraffin. Tissue sections were cut 4 µm thick and routinely stained with hematoxylin/eosin and specific Masson’s trichrome [56] to assess the density of collagen fibers. Mounted slides were examined and photographed using the Leica Application Suite (Leica Microsystems, Wetzlar, a light microscope). 

### 3.12. Morphometric Study 

The morphometric study was conducted using the Leica Qwin 500 Image Analyzer (LEICA Imaging Systems Ltd., Cambridge, UK) at the National Research Centre’s Pathology Department. The instrument consists of a Leica DM-LB microscope paired to a computer device (Leica Q 500 W) coupled with a JVC color video camera. The morphometric study was performed on Masson’s trichrome-stained slides. The measurements were taken from the microscope on a real-time image that was visualized on the screen. The area measurement was masked by a click on the positive stain reaction. The results immediately appeared on the screen in the form of a (μm^2^) area with a mean ± standard error [52].

### 3.13. Statistical Analysis

The data were represented as the means ± SEM. The data were exposed to one-way analysis of variance (ANOVA) followed by Tukey’s comparison test using the software program, GraphPad Prism (version 8.00). The level of significance was considered at *p* < 0.05.

## 4. Conclusions

The results of the study showed that isolated myricetin from *T. capensis* v. *aurea* successfully stimulates wound contraction as compared to the control group and other groups. These findings could justify the inclusion of this plant in the management of wound healing. Herein, the higher dose of myricetin possesses remarkable wound healing activities in rats that can be widely related to their ability to improve cellular re-epithelialization, cell proliferation, collagen deposition, and also as an effective anti-inflammatory agent for wound healing applications. Our findings showed the potent role of myricetin in treating chronic wounds, which is abundant in medicinal plants.

## Figures and Tables

**Table 1:** The change of wound area (mm^2^) and contraction percentage (%) in each group.

Group	Day 0	Day 7	Day 14
Wound	101.5 ± 1.1	82.5 ± 3.2 (18.7%) ^a^	33.1 ± 2.7 (67.3%)
Dermazin	115.3 ± 5.3	36.7 ± 1.6 * (63.8%)	2.7 ± 1.8 * (97.3%)
Paraffin	103.5 ± 2.7	54.5 ± 3.4 * (46.3%)	3.7 ± 2.2 * (96.3%)
10% Myricetin	113.8 ± 2.2	47.2 ± 6 * (53.4%)	11.0 ± 2.3 * (89.1%)
20% Myricetin	111.3 ± 2.8	34.1 ± 2.7 * (66.4%)	1.2 ± 0.9 * (98.8%)

^a^ Values are mean ± SEM (%), * significant at *p* < 0.05 compared with the wound group.

**Table 2:** Effect of myricetin treatment on serum levels of TNF-*α* and IL-1*β* in rats with the wounded model.

Groups	TNF-*α*	IL-1*β*
Normal control	76.2 ± 8.2	86.1 ± 7.7 ^a^
Wound	668.6 ± 49.2 *	616.3 ± 47.6 *
Dermazin	116.6 ± 13.9 **	131.4 ± 13.2 **
Paraffin	126 ± 14.2 **	148.9 ± 7.8 **
10% Myricetin	110.7 ± 12.9 **	136.3 ± 21.7 **
20% Myricetin	77.3 ± 3.8 **	96.3 ± 5.7 **

^a^ Results are presented as mean ± SEM (*n* = 6). Statistical analysis was performed using one-way ANOVA, where ** *p* < 0.05 versus wounded rats, * *p* < 0.05 versus normal control.

**Table 3:** Area of collagen (µm^2^) in the dermis of rats.

Group	Area of Collagen (µm²)
Normal	48 ± 4.5 ^a^
+ Control	28.7 ± 2.4 *
10% Myricetin	45.1 ± 5.9 **
20% Myricetin	54.8 ± 1.8 **
Drug	43 ± 5.5 **
Paraffin	25.5 ± 4.9 *

^a^ Data presented as mean ± SEM, * significant decrease at *p* < 0.05 as compared with normal, ** significant increase at *p* < 0.05 as compared with + control.

## Supplementary Materials

The following are available online, Figure S1: Figure S1. Macroscopic incision wound measurements of a skin section from (A) control positive group, (B) drug treated group (C) paraffin treated group, (D) 10% myricetin treated group, (E) 20% myricetin treated group.
