# Supplementary material for: Topical Wound Healing Activity of Myricetin Isolated from Tecomaria capensis v. aurea
Source: Molecules. 2020 Oct 22;25(21):4870. doi: 10.3390/molecules25214870 (PMC7659475; doi:10.3390/molecules25214870)
Supplement: Supplementary file 1 [file molecules-25-04870-s001.pdf]

molecules

Article

Topical Wound Healing Activity of Myricetin Isolated from *Tecomaria capensis* v. *aurea*

Abdelsamed I. Elshamy[1*], Naglaa M. Ammar[2], Heba A. Hassan[2], Walaa A. El-Kashak[1], Salim S. Al-Rejaie[3], Ahmed M. Abd-ElGawad[4,5*], Abdel-Razik H. Farrag[6]

1 Chemistry of Natural Compounds Department, National Research Centre, 33 El Bohouth St., Dokki, Cairo, 12622, Egypt; ai.el-shamy@nrc.sci.eg
2 Therapeutic Chemistry Department, National Research Centre, 33 El Bohouth St., Dokki, Cairo, 12622, Egypt; nm.ammar@nrc.sci.eg
3 Department of Pharmacology & Toxicology, College of Pharmacy, King Saud University, Riyadh 11451, Saudi Arabia; rejaie@ksu.edu.sa
4 Plant Production Department, College of Food & Agriculture Sciences, King SaudUniversity, P.O. Box 2460, Riyadh 11451, Saudi Arabia; aibrahim2@ksu.edu.sa
5 Department of Botany, Faculty of Science, Mansoura University, Mansoura 35516, Egypt; dgawad84@mans.edu.eg
6 Departments of Pathology, National Research Centre, 33 El Bohouth St. Dokki, Cairo 12622, Egypt; ar.hussein@nrc.sci.eg
* Correspondence: ai.el-shamy@nrc.sci.eg; Tel.: +201005525108 (A.I.E.), aibrahim2@ksu.edu.sa; Tel.: +966-562680864 (A.M.A-E.)

Received: 27 September 2020; Accepted: 19 October 2020; Published: 22 October 2020

Supplementary Materials

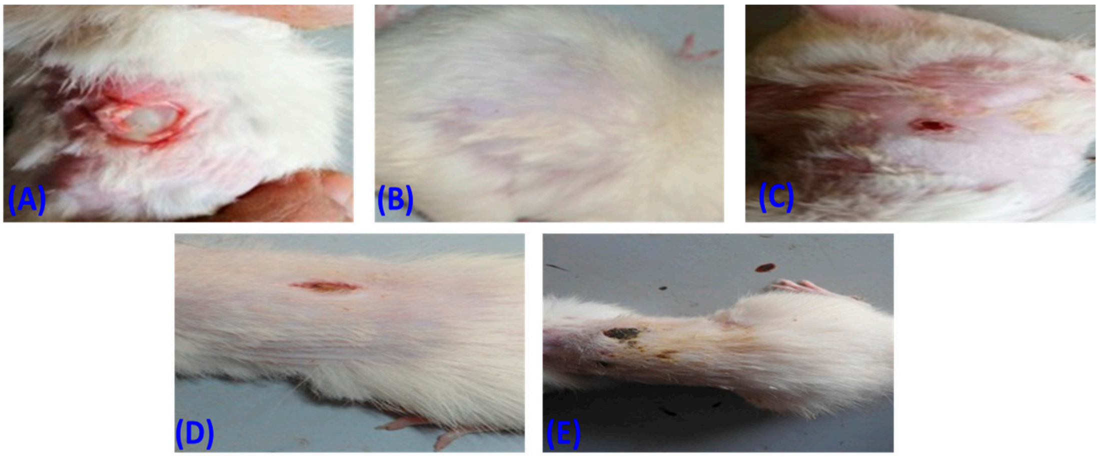

Figure S1. Macroscopic incision wound measurements of a skin section from A) control positive group, B) drug-treated group C) paraffin treated group, D) 10% myricetin treated group, E) 20% myricetin treated group.
